# Solar dimming by increased moisture in a warmer world

**DOI:** 10.1093/nsr/nwaf038

**Published:** 2025-02-08

**Authors:** Richard P Allan

**Affiliations:** Department of Meteorology and National Centre for Earth Observation, University of Reading, UK

Surface solar radiation drives the global water cycle and provides energy for photosynthesis as well as solar power. A decline in sunlight reaching the surface of Earth has previously been identified and linked with greater reflection and absorption by aerosol particle pollution and the influence of this haze on cloud [1]. While cleaner air has led to a recovery in the surface sunlight that is received in some regions such as the USA and Europe, and more recently China [1,2], a new study has identified a growing influence of greenhouse-gas-induced warming and a consequent increase in atmospheric moisture [3]. By combining the latest observations and state-of-the-art climate simulations, the new analysis unpicks the causes of a decline in sunlight reaching the surface of Earth. Previous predictions based on computer modelling [4] are confirmed, while ongoing changes in surface insolation are shown to depend on future emissions scenarios that determine how much warmer and moister the atmosphere will become.

**Figure 1.**
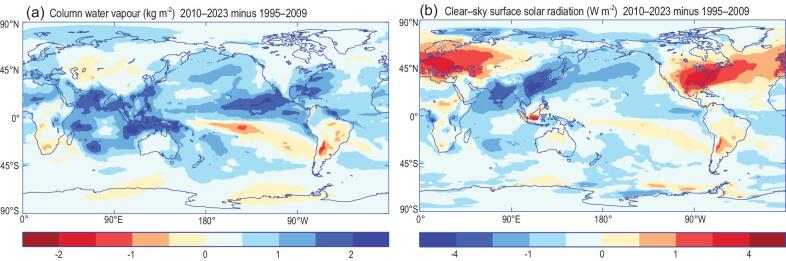
Changes in (a) atmospheric column-integrated water vapour and (b) surface clear-sky downward solar radiation (1995–2009 to 2010–2023) simulated by the European Centre for Medium-range Weather Forecasts 5th generation reanalysis (ERA5) [11].

Water vapour is increasing with atmospheric warming [5] and this is intensifying the global water cycle [6]. Rising water vapour concentrations are also amplifying warming through radiative feedback, which is dominated by the potent greenhouse trapping of infrared radiation but compounded by greater absorption of sunlight by more numerous water molecules [7]. This latter effect steals sunlight that is bound for the surface of Earth. Recent increases in column-integrated water vapour are prevalent globally, particularly over the ocean (Fig. 1a). Concurrent decreases in surface downward clear-sky solar radiation are also evident over the tropics, particularly in the west Pacific and Indian Ocean, while regions of decreasing water vapour over parts of the east Pacific and tropical Africa and South America correlate spatially with increases in clear-sky surface solar radiation (Fig. 1b). However, large increases in surface clear-sky insolation over the USA and Europe are instead explained by the declining absorption and scattering of sunlight by aerosols [1,2], while continued increases in air pollution over India are associated with reduced surface sunlight (Fig. 1b), which compound the extra absorption of sunlight from more atmospheric moisture.

The new analysis [3] is the first to clearly attribute a growing component of the observed changes in surface sunlight to atmospheric moistening and, further, to highlight how future changes in sunlight received at the ground are sensitive to greenhouse-gas and aerosol-emissions scenarios. The growing impact of atmospheric moistening on declining surface sunlight in recent decades is shown to continue in the higher greenhouse-gas-emission scenarios. Yet, the increasing atmospheric moisture only marginally limits the sunlight that reaches the surface and much of the extra energy that is absorbed by the atmosphere ultimately heats the surface. Therefore, increasing atmospheric water vapour strongly amplifies the warming of climate.

Recent and future regional changes in cloud, aerosols and water vapour are uncertain. Moisture variation can be spurious in reanalyses due to changing observing systems [5] while coupled climate models overestimate warming and atmospheric moistening in the early 2000s [5] and are unable to capture an observed drying in arid and semi-arid regions [8]. The complex interplay between aerosols, cloud and atmospheric moisture implies substantial regional variation and uncertainty. Cloud changes are not well constrained and are influenced by changing wind patterns that may dominate signals of an increase or decrease in solar radiation in many regions. Therefore, the water-vapour-induced decreases in surface insolation may be limited compared with other regional factors that involve cloud and aerosols. A recent observed increase in the planetary absorption of sunlight [9,10] that is not well understood adds to the debate surrounding how the energy budget of Earth is changing. Ground-truth observations are therefore crucial in determining how the amount of sunlight that reaches the surface of Earth will alter in the future.
